# Sport-specific demands on sustained attention in elite athletes: a novel within-subjects approach for investigation

**DOI:** 10.3389/fspor.2026.1765258

**Published:** 2026-03-13

**Authors:** Michelle J. Blumberg, Michael Esterman, Kathryn Johnston, Nick Wattie, James Brough, Ryan Atkison, Joseph Baker, Magdalena Wojtowicz

**Affiliations:** 1 Department of Psychology, York University, Toronto, ON, Canada; 2 National Center for PTSD, VA Boston Healthcare System, Boston, MA, United States; 3 Department of Psychiatry, Boston University Chobanian and Avedisian School of Medicine, Boston, MA, United States; 4 Tanenbaum Institute for Science in Sport, Faculty of Kinesiology and Physical Education, University of Toronto, Toronto, ON, Canada; 5 Faculty of Health Sciences, Ontario Tech University, Oshawa, ON, Canada; 6 Canadian Sport Institute Ontario, Toronto, ON, Canada

**Keywords:** cognitive functioning, high performance, intraindividual variability, sport, sustained attention, variance time course

## Abstract

**Background:**

Research has primarily examined between-group differences in athletes’ cognition, and particularly sustained attention. More nuanced approaches are needed to better characterize athletes’ attentional abilities.

**Objective:**

To examine associations between sport type and traditional Sustained Attention to Response Task (SART) indices, and to apply a novel method to characterize within-person fluctuations in sustained attention performance.

**Methods:**

Participants were 198 elite athletes from a regional sport training and development organization in Toronto, Canada (*M*_age_ = 17.67 years, *SD* = 4.58; 56.57% female). Linear regressions explored associations between sport type (team, speed-strength, precision-skill) and SART indices (speed, accuracy, variability), controlling for age and sex. The variance time course procedure was then used to examine intraindividual fluctuations in sustained attention and to explore whether different patterns of performance emerge across sport types.

**Results:**

Using a pre-established sport classification system, team sport athletes were significantly more accurate on the SART than speed-strength (*B* = −0.81, *p* = .002) and precision-skill (*B* = −0.45, *p* = .016) athletes; however, the groups did not differ in speed or variability. The variance time course procedure showed that athletes fluctuated between ‘in the zone’ and ‘out of the zone’ attentional states, with ‘out of the zone’ performance marked by greater variability and more error-prone responding. We also provide preliminary evidence that patterns of performance variability differ across sport types.

**Conclusion:**

Different sports may place distinct demands on specific dimensions of attentional performance. Within-person approaches are warranted to advance the field toward more precise assessment of athletes’ cognitive functioning.

## 1 Introduction

In addition to physical abilities and technical skills, cognition is believed to play an important role in sport performance and athletic success. Compared to non-athletes, athletes have been shown to exhibit superior performance on cognitive tests measuring aspects of attention, executive functioning, and processing speed (1–3). Moreover, higher sport expertise among athletes has been associated with higher scores on tests of cognitive functioning (4–6). The cognitive domain of attention, broadly defined as the allocation of cognitive resources to internal or external stimuli, plays a crucial role in sport (2, 7, 8). This may be especially true in the context of team sports, where athletes must simultaneously monitor the actions and positions of multiple players and sport-related stimuli (9). Most research on attention in sport has centered on more transient sub-processes, including alternating, selective, and divided attention (9). Sustained attention, a sub-process involving the ability to focus goal-directed attention over extended periods of time, may be particularly important for sport performance (8). Specifically, the capacity to maintain focus on a particular stimulus or location in the sporting environment, while ignoring distractions for prolonged periods of time, is essential in both team and individual sports (9). Numerous other cognitive processes important for sport, including learning, memory, and executive functioning, also rely on sustained attention (10, 11), highlighting the importance of investigating athletes’ sustained attention abilities. Despite support for the notion that sustained attention is important for sport, there is a paucity of research examining indices of sustained attention in athlete populations.

Studies examining sustained attention in athletes have primarily focused on vigilance, a term often used synonymously with sustained attention, though it reflects a narrower aspect of sustained attention. Defined as the capacity to respond accurately to target stimuli (12), vigilance has been shown to be superior in athletes compared to their non-athletic counterparts. For instance, highly fit children (i.e., as measured by a range of cardiovascular tests) showed fewer errors of omission in a flanker task than less fit children (13). Although the flanker task is not a traditional measure of sustained attention, omission errors are classically viewed as an index of failures in sustained attention. Luque-Casado et al. (14) showed superior performance on the Psychomotor Vigilance Task (PVT), as evidenced by faster response times (RTs), in highly fit young adults compared to their less fit counterparts. Similarly, Ballester et al. (15) found that overall RTs and lapses on the PVT were significantly lower and more resistant to the effects of time spent on task in individuals who played soccer compared to non-athletes. Another study examined whether martial arts athletes could outperform novice athletes on the continuous performance task (CPT) and found no differences in accuracies or RTs; however, differences in brain electrical activity were found between the groups (16, 17). These results suggest behavioural indices in vigilance or sustained attention tests (measured in accuracy and/or RTs) may not always be sufficiently sensitive to detect differences in sustained attention between athletes and non-athletes. Other studies examining aspects of sustained attention provide preliminary support for the notion that expert athletes may exhibit superior sustained attention abilities than their less athletic peers (18). These studies provide support for the cognitive component skills theory, which posits that sport represents an environment for vigilance training, resulting in more efficient brain networks and attentional skills (6). Most studies, however, do not distinguish between the impact of generic sport participation and the specific effects of different sports on vigilance performance. Further research comparing the effects of sport types on more sensitive indices of vigilance is required.

The majority of studies examining sustained attention fail to acknowledge that performance fluctuates from moment-to-moment within an individual, and that these fluctuations impact overall task performance (19). To examine fluctuations in sustained attention, researchers typically look at variability on repeated trials of an RT task, often conceptualized as intraindividual variability (IIV) (20, 21). Historically, IIV has been dismissed as error, instability, or noise, while measures of central tendency (i.e., mean RT) have dominated neuropsychological research (22). This has been especially evident in studies of healthy athlete cognition. More recently, however, it has been proposed that IIV represents an indicator of cognitive functioning that is independent of overall mean performance (23–27). Moreover, individuals who perform well on cognitive tasks tend to show less variability than those who perform poorly (25, 28). Measures of RT variability, such as IIV, may better capture an individual's ability to sustain attention compared to mean-level performance measures (29). Critically, IIV in sustained attention remains underexplored in athlete populations.

A number of factors have been identified as possible contributors to performance variability (30). For instance, age is frequently associated with increased IIV on RT tasks. IIV across the lifespan is characterized by a U-shaped curve, such that childhood and older age are associated with greater IIV (28, 31). As youth age, variability in performance decreases, reflecting the development of better attentional abilities (32). Moreover, variability in performance on cognitive tasks may be an indicator of Central Nervous System (CNS) dysfunction, and therefore IIV may be a useful marker of cerebral integrity (33, 34). Evidence for the relationship between IIV and CNS dysfunction comes from studies demonstrating that variability in cognitive test performance is associated with conditions characterized by neurological disturbance, such as traumatic brain injury (23, 24), multiple sclerosis (35, 36), attention-deficit/hyperactivity disorder (37), and dementia (38). Importantly, greater IIV in cognitive performance has been observed in the earlier stages of neurodegenerative conditions, while mean-level performance may not reveal the presence of disease (39, 40). Thus, IIV may represent a more sensitive indicator of cerebral integrity that provides unique information about cognitive functioning beyond that provided by indices of average performance (41).

Measures of IIV can also help elucidate the cerebral integrity of seemingly healthy individuals. Involvement of frontal networks has been a key focus in the IIV literature (40). Specifically, smaller prefrontal white matter volumes (42) and frontal white matter hyperintensities (43) have been associated with increased IIV among presumably healthy adults (40). Moreover, in healthy adult populations, greater IIV has been weakly associated with worse overall task performance, suggesting IIV may reflect aspects of task performance not fully captured by traditional performance measures, such as mean RT (29). Thus, IIV in cognitive performance may also be a useful index of cognitive functioning in healthy populations, and particularly among athletes for whom momentary lapses in attention can determine success or failure in their sport.

Several limitations exist within the current sustained attention literature. To our knowledge, the only study that has examined variability in sustained attention in healthy athletes (44) examined whether IIV differed between strategic-sport (soccer) and static-sport (track and field) athletes, finding no differences between the groups. Relatedly, analyses of IIV have been limited to differences across groups or task conditions, and therefore, have yet to explore within-subject variability as sustained attention varies over time (21). Furthermore, most studies examining IIV in sustained attention have employed Go/No-Go tasks, such as the CPT and the Sustained Attention to Response Task (SART). The limitation of these tasks is that they involve abrupt stimulus onsets and offsets, which function as exogenous cues that signal trial-to-trial stimulus changes (10). Consequently, they may not represent pure measures of sustained attention. New methods for assessing sustained attention are required to better characterize moment-to-moment fluctuations in attention. This is particularly true for athlete populations where sustaining focus is crucial for sport performance and athletic success.

Recently, a novel method was developed to provide a more nuanced approach to characterizing sustained attention. Specifically, Esterman et al. (21) developed a variation of the traditional Go/No-Go CPT, the gradual onset CPT (gradCPT), to reduce the abrupt trial-based nature of these previous tasks. Using the gradCPT, the variance time course (VTC) procedure was developed as a method for examining trial-by-trial RT variability (10, 19, 21, 28, 45–49). VTC analyses have demonstrated that attention fluctuates between periods of low variability, high accuracy, and small error-prone responses (i.e., ‘in the zone’), and periods of higher variability, lower accuracy, and more error-prone responses (i.e., ‘out of the zone’) (see Figure 2 in Esterman et al. (21) for an example). VTC analyses have primarily been conducted using variations of the gradCPT and in healthy adult populations. Accordingly, the VTC is a relatively unexplored concept that has not been used to examine moment-to-moment fluctuations in sustained attention in athletes.

Taken together, novel methods for characterizing the sustained attention abilities of elite athletes are warranted, as sustained attention underlies numerous other cognitive functions (10), and in turn, athletic success. Accordingly, the overarching goal of the current study was to evaluate the utility of a novel method for more precise characterization of the sustained attention abilities of athletes. Given that sustained attention has been underexplored within athlete populations and across different sport types, we first examined the association between sport type (i.e., team, precision-skill, speed-strength) and traditional indices of sustained attention (i.e., speed, accuracy, variability) in a sample of elite athletes. It was hypothesized that team sport athletes would outperform speed-strength and precision-skill athletes across all three indices of sustained attention. Next, we sought to examine whether a VTC procedure can be applied to a traditional sustained attention task (the SART) in an elite athlete population. We first examined whether we could replicate the findings of Esterman et al. (21) using the SART. We then explored how the VTC procedure could be used to better characterize moment-to-moment fluctuations in sustained attention in individual athletes, and whether these fluctuations in sustained attention differ across sport types.

## 2 Materials and methods

### 2.1 Study design and participants

Two hundred and twenty-four athletes were recruited as part of a larger observational cross-sectional study conducted at the Canadian Sport Institute Ontario (CSIO), a regional high-performance sport training and development organization. Participants were categorized into three sport types based on criteria developed by McKay et al. (50): team, speed-strength, and precision-skill. Team sports are described as object sports where the primary objective is to manipulate an object while another competitor is in direct confrontation (e.g., American football, basketball, field hockey, soccer). Speed-strength sports are individual sports where one competitor cannot directly interfere with the performance of another (e.g., Alpine skiing, speed skating, sprinting, weightlifting). Precision-skill sports represent a combination of individual and object (i.e., requiring an object for task execution) sports which place a larger emphasis on skill rather than physiology or strength as a determinant of success (e.g., Curling, diving, figure skating, golf). The distribution of sports by sport type is presented in Table 1. All participants provided written informed consent. The study was approved by the lead researcher's institution's research ethics board (York University Research Ethics Board #e2022–343).

**Table 1:** Distribution of sports by sport type (*N* = 198).

Sport	*N*
Team Sports	80
Field hockey	1
Basketball	8
Ice hockey	17
Rugby (Includes 7's)	20
Volleyball (Indoor and beach)	34
Speed-Strength Sports	34
Cycling	14
Cross country skiing	5
Athletics	8
Swimming	4
Canoe-Kayak	2
Rowing	1
Precision-Skill Sports	84
Curling	20
Sailing	4
Diving	17
Artistic swimming	15
Figure skating	3
Ski moguls	3
Freestyle ski	21
Alpine ski	1

### 2.2 Materials and procedure

As part of the larger study conducted at the CSIO, participants completed a series of demographic and self-report questionnaires. Relevant to the current study, participants provided information regarding their age, sex, and sport. Participants then completed a computerized cognitive assessment administered on a laptop called Creyos cognitive assessment, previously called Cambridge Brain Sciences (51).

#### 2.2.1 Sustained attention to response task (SART)

The SART is a continuous performance task commonly used to assess attention and response inhibition over longer periods of time. The SART was designed to capture everyday slips in attention during routine tasks. The SART was conducted using the Creyos cognitive assessment software. The task displays 225 trials with a random number from 1 to 9, followed by an asterisk. Participants are instructed to press the spacebar as quickly as possible when the asterisk turns bold (i.e., Go trials), unless the number 3 appeared (i.e., No-Go trials). More specifically, they are instructed to not respond when the asterisk turns bold after the number 3 appeared and to wait until the next number appears. Since the number 3 only appears for 25 of the 225 trials, participants become accustomed to automatically responding after every number. Accordingly, slips in attention are evident when participants incorrectly respond to No-Go trials (i.e., commission error) or when they fail to respond to a Go trial (i.e., omission error). Participants are told that speed and accuracy are equally important for successful performance on the task. The task takes approximately six minutes to complete and requires participants to maintain sustained attention for the entire duration of the task.

No-Go trials (i.e., the number 3) are presented in a pseudo-random order, with some consistency across participants: each 45-trial block is made up of: (A) an 18-trial sub-block with two of each number (including the number 3), pulled in random order; (B) another 18-trial sub-block with two of each number (including the number 3), pulled in random order; and (C) a 9-trial sub-block with one of each number (including the number 3), pulled in random order. Sub-blocks A, B, and C are in random order for each of the five 45-trial blocks in the task.

SART stimuli are presented for 313 ms. The inter-stimulus window time is 1,313 ms from the start of one stimulus until the next, which overlaps with the response window time where responses are recorded at any time after the stimulus is presented, but only once the response cue (i.e., bolded asterisk) appears. Thus, the stimulus appears for 313 ms, followed by the asterisk for 125 ms, then the response cue for 875 ms. SART digits range in font size (i.e., 48 point, 72 point, 94 point, 100 point, or 120 point), and therefore, the visual angle will vary between stimuli. All participants completed the SART on a standard 13” laptop and were seated approximately 50–60 cm away from the screen. As such, the visual angle varied between approximately 0.94° to 1.12° for digits presented in 48 point font; 1.41° to 1.69° for digits presented in 72 point font; 1.84° to 2.20° for digits presented in 94 point font; 1.95° to 2.34° for digits presented in 100 point font; and 2.34° to 2.81° for digits presented in 120 point font. It is important to note that the SART can be completed on a variety of devices and screen sizes. As a result, visual angles will vary across studies.

### 2.3 Statistical analysis

All statistical analyses were performed in R Version 4.1.1 (52). Participants were excluded if their accuracy rates for Go trials on the SART were less than 50% (*N* = 19).

#### 2.3.1 Computing traditional sustained attention indices

For each participant, mean RT was computed by taking the average RT across correct Go trials. To quantify accuracy on the SART, d prime (d’) was calculated, a measure of a participant's ability to discriminate targets (Go trials) from nontargets (No-Go trials). d’ is calculated from the hit rate and false-alarm (FA) rate using the formula, d’ = Z_Hit_ – Z_FA_ (67). For participants who committed no errors (i.e., hit rate of 1 and FA rate of 0), d’ is infinite. To avoid infinite d’ values for participants with perfect accuracy, we applied a standard signal-detection correction, in which hit rates of 1 were replaced with 0.999 and FA rates of 0 were replaced with 0.001 (53). For each participant, IIV was examined by computing the coefficient of variation (CoV) using the formula, CoV = Intraindividual Standard Deviation (ISD)/Mean RT.

#### 2.3.2 Associations between sport type and sustained attention indices

Next, in a series of multiple linear regressions, sport type (team, speed-strength, precision-skill) was used to predict sustained attention indices (mean RT, d’, CoV), while controlling for age (years) and sex (male:female). First, to examine whether sustained attention indices differed between team sport athletes and speed-strength or precision-skill athletes, sport type was dummy coded with team as the reference group. Then, to explore whether sustained attention indices differed between speed-strength and precision-skill athletes, sport type was dummy coded with precision-skill as the reference group. Assumptions of normality and homoscedasticity of the residuals were assessed via Q-Q plots and residual-vs.-fitted plots, respectively, and were determined to be acceptable. Using Cook's distance, no cases were deemed to be influential cases.

#### 2.3.3 Computing the variance time course

Beyond traditional indices of sustained attention, an exploratory aim of this study was to characterize trial-by-trial RT fluctuations using the VTC procedure. Trials with no responses (i.e., correct No-Go trials, omission errors on Go trials) and outlier trials (i.e., trial RTs greater or less than three standard deviations from the mean RT) were interpolated linearly, such that missing values were linearly estimated from RTs of the surrounding two trials. For each participant, VTCs were computed from all 225 trials of the SART, including linearly imputed trials. RTs for each trial were Z-transformed within-subject to normalize the scale of the VTC. The absolute value of the Z scores was then taken, so that the value assigned to each trial represented the absolute deviation of the trial's RT from the mean RT of the task. Deviant RTs, whether fast or slow, were thought to represent reduced attention to the task: fast RTs may indicate premature responding and inattention to the possible need for response inhibition, while slow RTs indicate reduced attention to or inefficient processing of the stimuli, requiring more time to respond to Go trials (21, 54). From the absolute Z scores, a smoothed VTC was computed using a centered Gaussian kernel spanning 19 trials, such that each point reflects a weighted moving average over the current trial and the surrounding nine trials on either side. Since this approach requires complete kernel overlap, smoothed values are undefined at the beginning and end of the task. As a result, the first and last nine trials were omitted from the VTC. Finally, we calculated the median of each participant's smoothed VTC. Trials below the median were considered to be ‘in the zone’, while trials equal or above the median were considered ‘out of the zone’.

Using paired samples t-tests, we compared the errors rates (commission and omission errors) and IIV (CoV) of ‘in the zone’ and ‘out of the zone’ trials to ensure we could replicate previous VTC analyses done using the gradCPT (21). Note that omission errors occurred rarely (average omission rate of 4.83%); however, we report omission errors for completeness. We also computed the average RT of trials that preceded a hit (i.e., correct Go trial), correct omission (i.e., correct No-Go trial), commission error (i.e., responding to a No-Go trial), and omission error (i.e., failing to respond to a Go trial), and using paired-samples t-tests, we examined whether this differed when participants were in the zone vs. out of the zone. As a sensitivity analysis, we re-ran these comparisons by sport type and observed the same pattern of results. Therefore, we present the results for the full sample.

Finally, we explored whether we could use the VTC procedure to examine whether sustained attention depletes over time. To do so, we compared the proportion of trials in the zone across the five trial bins described above (i.e., the SART is constructed in five blocks of 45 trials). Since the first and last nine trials were omitted during the smoothing process, bins one and five contained 36 trials while bins two through four contained 45 trials. A repeated-measures Analysis of Variance was conducted to test whether there was a main effect of trial bin on the proportion of trials in the zone for the entire sample and then for the three sport type groups (i.e., team, speed-strength, precision-skill). Where a significant main effect of trial bin was observed, Bonferroni-corrected paired samples t-tests were conducted to examine differences between trial bins.

## 3 Results

Of the 224 participants who completed the SART, 198 were included in the analyses. Participants were excluded if their accuracy rates for Go trials were lower than 50% (*N* = 19) or if they were missing age, sex or sport data (*N* = 7). The mean age of the sample was 17.67 years (range = 10–39, *SD* = 4.58) and 56.57% of the sample was female. There were 80 team sport athletes, 34 speed-strength athletes, and 84 precision-skill athletes.

### 3.1 Associations between sport type and sustained attention indices

A series of multiple linear regressions were conducted to examine whether sport type (i.e., team, speed-strength, precision-skill) predicted indices of sustained attention (mean RT, d’, CoV), while controlling for age and sex. The overall model predicting mean RT was not significant, *F*(4, 193) = 0.02, *p* = .999, *R*^2^ = .001. Mean RT did not significantly differ between team sport athletes and speed-strength (*B* = 5.62, *p* = .795) or precision-skill (*B* = 4.13, *p* = .795) athletes. Similarly, when the precision-skill group was coded as the reference group, no significant differences in mean RT were found between speed-strength and precision-skill athletes. Age and sex were not significantly associated with mean RT.

The overall model predicting accuracy was significant, *F*(4, 193) = 7.78, *p* < .001, explaining approximately 14% of the variance in d’. Compared to team sport athletes, both speed-strength (*B* = −0.81, *p* = .002) and precision-skill (*B* = −0.45, *p* = .016) athletes demonstrated significantly lower accuracy as measured by d’. When the precision-skill group was coded as the reference group, no significant differences in d’ were found between speed-strength and precision-skill athletes. Age was significantly associated with accuracy, such that as athletes age, their accuracy on the SART improved (*B* = 0.09, *p* < .001).

Finally, the overall model predicting intraindividual variability was significant, *F*(4, 193) = 4.16, *p* = .003, explaining approximately 8% of the variance in CoV. CoV did not significantly differ between team sport athletes and speed-strength (*B* = 0.07, *p* = .092) or precision-skill (*B* = 0.01, *p* = .820) athletes. Similarly, when the precision-skill group was coded as the reference group, no significant differences in CoV were found between speed-strength and precision-skill athletes. Age was significantly associated with CoV, such that as athletes age, variability in sustained attention performance decreased (*B* = −0.01, *p* = .002). The results of the regression models are summarized in Table 2. The results of the regression models with the precision-skill group coded as the reference group are summarized in a Supplemental Table.

**Table 2:** Predicting indices of sustained attention based on sport type.

Variable	*B*	*SE (B)*	*t*	*p*	95% CI for *B*
Model 1: Mean RT					
Age	−0.10	1.59	−0.06	.949	[−3.24, 3.04]
Sex: Female	2.07	14.86	0.14	.890	[−27.25, 31.38]
Sport Type: Speed-Strength	5.62	21.65	0.26	.795	[−37.08, 48.33]
Sport Type: Precision-Skill	4.13	15.87	0.26	.795	[−27.17, 35.43]
Model 2: d’					
Age	**0.09**	**0.02**	**4.83**	**<.001**	**[0.05, 0.13]**
Sex: Female	0.08	0.17	0.47	.637	[−0.26, 0.42]
Sport Type: Speed-Strength	**−0.81**	**0.25**	**−3.22**	**.002**	**[−1.31, −0.31]**
Sport Type: Precision-Skill	**−0.45**	**0.18**	**−2.43**	**.016**	**[−0.81, −0.08]**
Model 3: CoV					
Age	**−0.01**	**0.003**	**−3.16**	**.002**	**[−0.02, −0.004]**
Sex: Female	−0.06	0.03	−1.95	.052	[−0.11, 0.001]
Sport Type: Speed-Strength	0.07	0.04	1.70	.092	[−0.01, 0.15]
Sport Type: Precision-Skill	0.01	0.03	0.23	.820	[−0.05, 0.07]

Model 1: *F*(4, 193) = 0.02, *p* = .999, *R*^2^ = .001; Model 2: *F*(4, 193) = 7.78, *p* < .001, *R*^2^ = .14; Model 3: *F*(4, 193) = 4.16, *p* = .003, *R*^2^ = .08. RT, reaction time; CoV, coefficient of variation.

Bold values indicate a significant relationship between the variable and outcome.

### 3.2 Examining the variance time course

**Figure 1:**
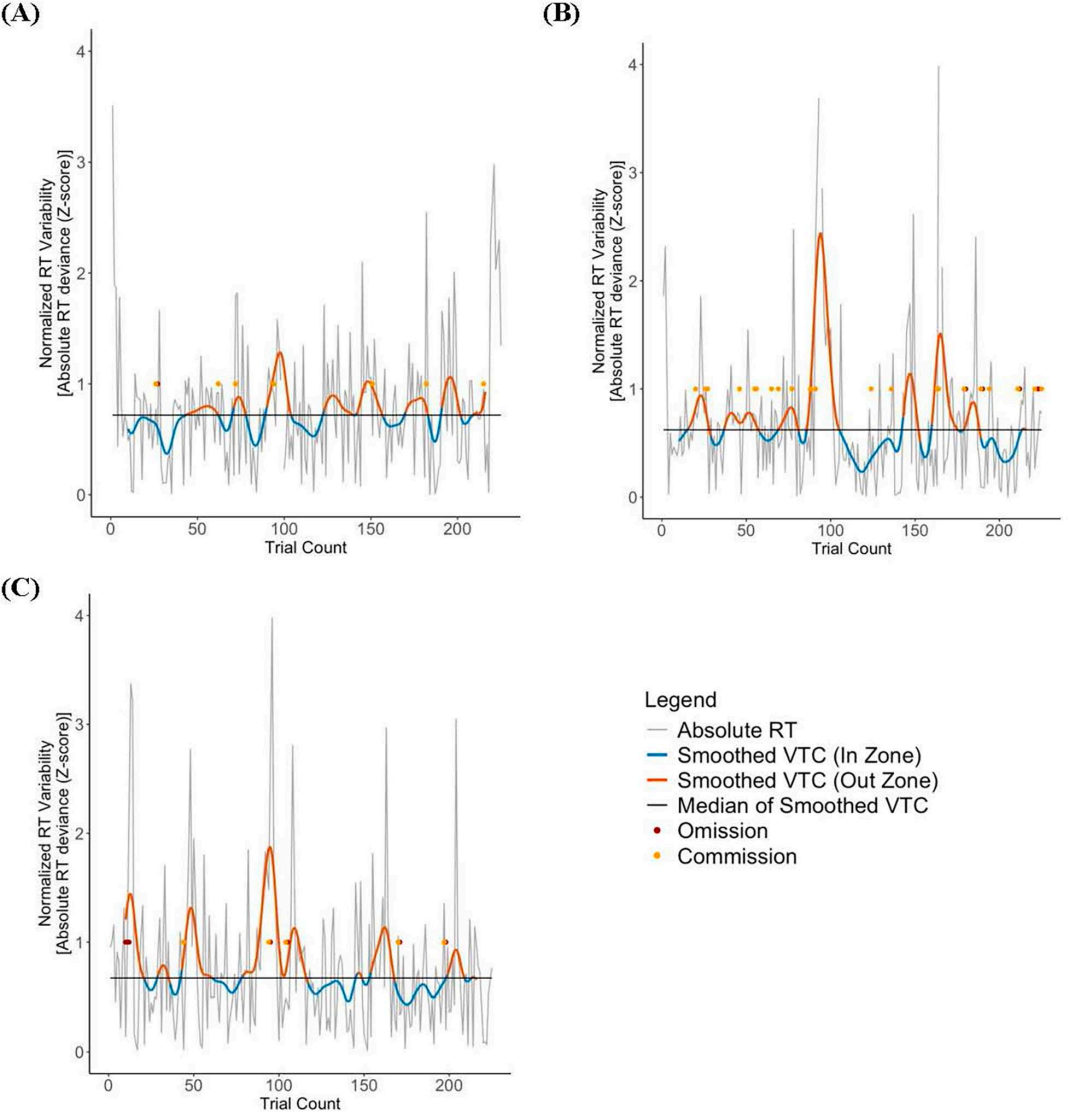
An example of a VTC for three participants. Performance is considered ‘in the zone’ when the smoothed VTC curve is below the participant's median RT; performance is considered ‘out of the zone’ when the smoothed VTC curve is equal or above the participant's median RT. **(A)** A representative team sport athlete, **(B)** A representative speed-strength athlete, **(C)** A representative precision-skill athlete.

We then examined whether the VTC procedure can be applied to performance on the SART in a sample of elite athletes. An example of a VTC for three representative participants is depicted in Figure 1 A) Team sport athlete, B) Speed-strength athlete, C) Precision-skill athlete. Specifically, the participants’ absolute deviation from their median RT is plotted as a function of trial count. Commission and omission errors can be visualized against the participant's RT variability.

**Figure 2:**
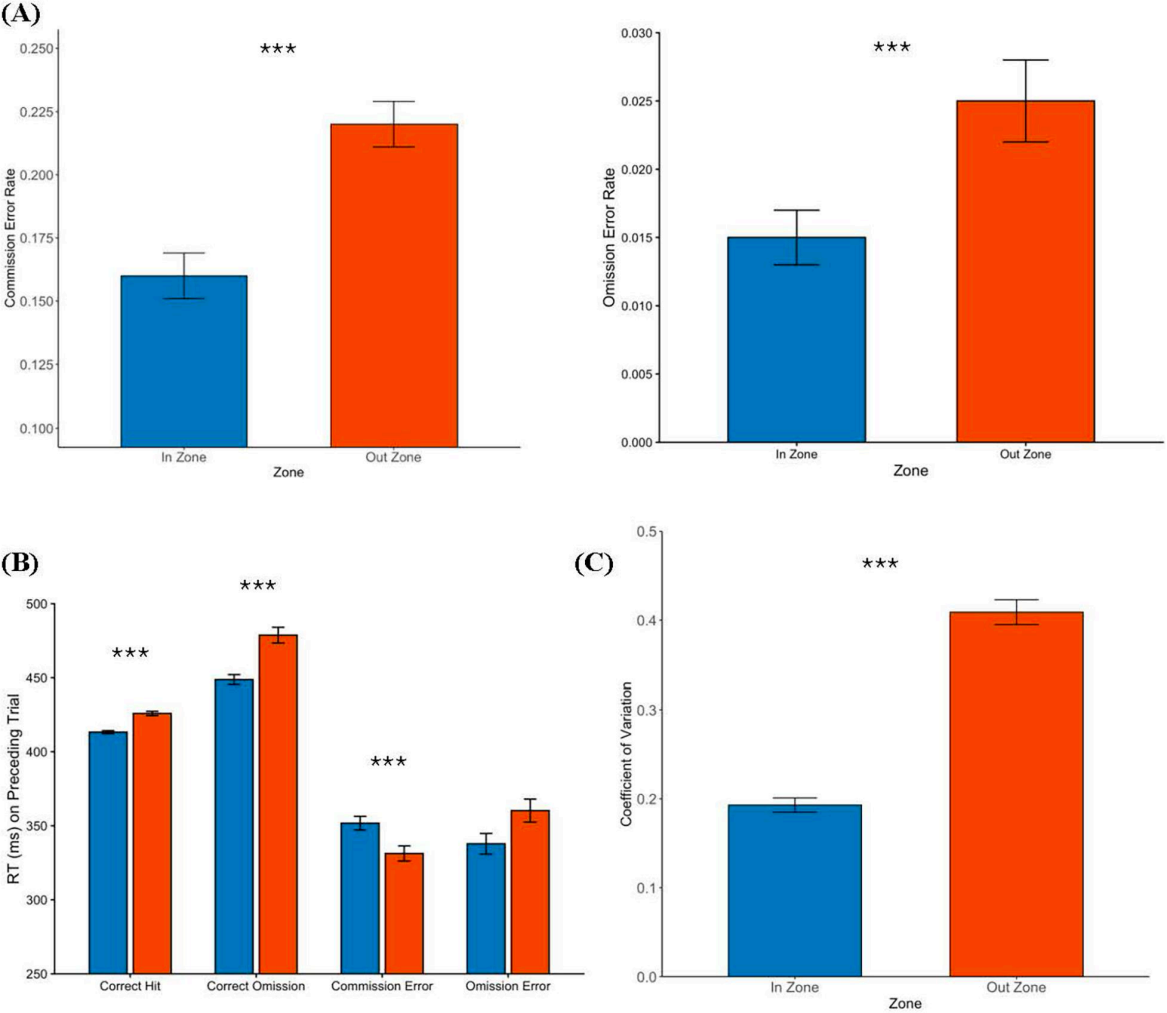
Sustained attention as a function of attentional state (in the zone vs. out of the zone). **(A)** Participants made more errors of commission and omission when out of the zone than when in the zone. **(B)** Participants were significantly slower on trials preceding correct trials when out of the zone than when in the zone. Participants were significantly faster on trials preceding commission errors when out of the zone than when in the zone. In contrast, participants were significantly slower on trials preceding omission errors when out of the zone. **(C)** Performance was significantly more variable when out of the zone than when in the zone. *Significant at.05 level; **at.01 level or lower; ***at.001 level or lower.

Participants committed significantly more commission, *t*(197) = 6.87, *p* < .001, and omission errors, *t*(197) = 5.48, *p* < .001, when out of the zone than when in the zone (see Figure 2A). Participants were significantly slower on trials preceding hits when out of the zone than when in the zone, *t*(197) = −4.23, *p* < .001. Participants were also significantly slower on trials preceding correct omissions when out of the zone than when in the zone, *t*(191) = −3.85, *p* < .001. In contrast, participants were significantly faster on trials preceding commission errors when out of the zone than when in the zone, *t*(172) = 5.19 *p* < .001. RTs of trials preceding omission errors did not differ between out of the zone and in the zone trials, *t*(84) = 0.71, *p* = .481, possibly reflecting limited power due to the occurrence of very few omission errors across the sample. Figure 2B plots the RT of trials preceding hits, correct omissions, commission errors, and omission errors, by zone. Finally, participants were significantly more variable, as measured by CoV, when out of the zone than when in the zone *t*(197) = 26.86, *p* < .001 (see Figure 2C).

**Figure 3:**
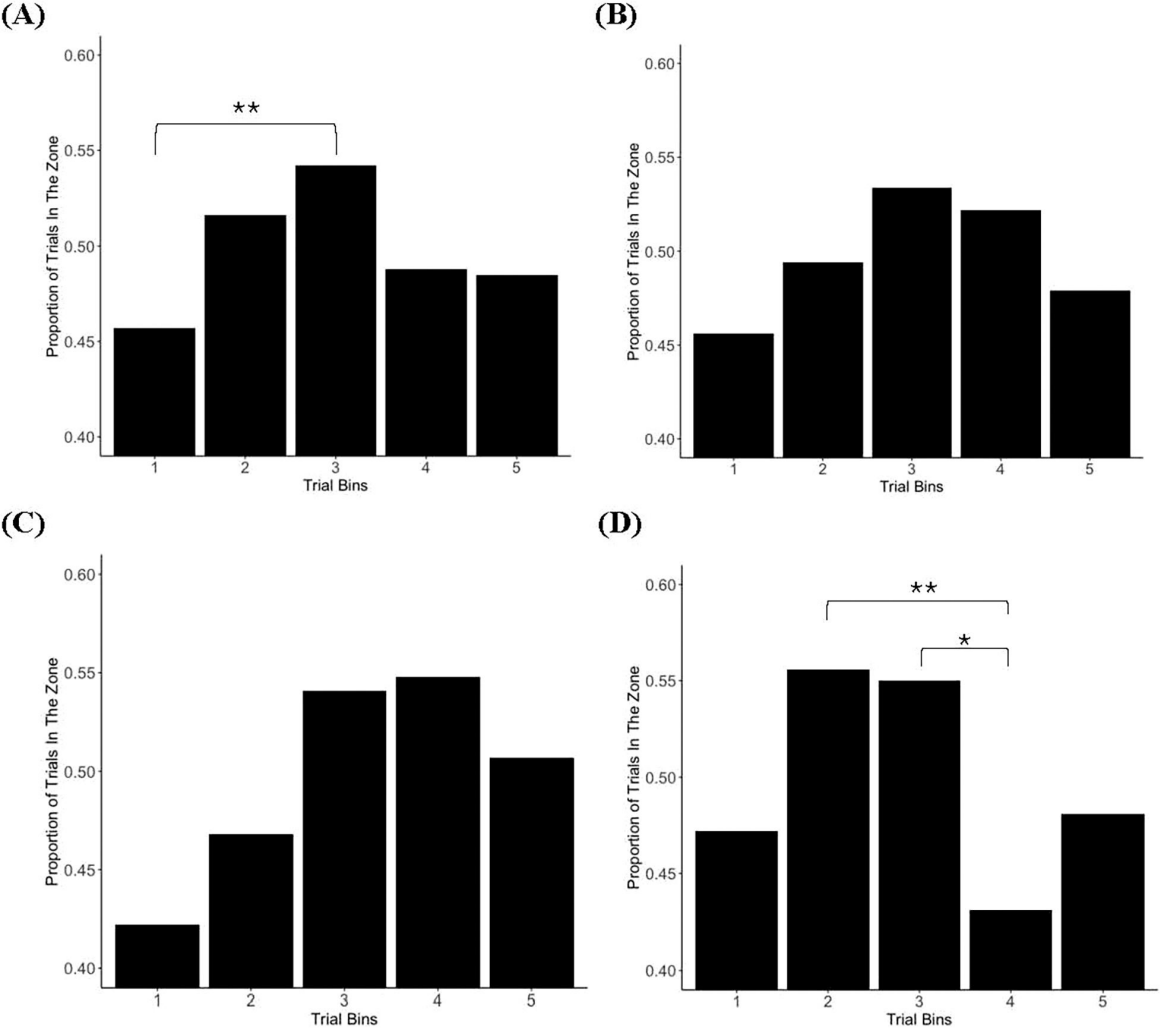
Proportion of trials in the zone by trial bin. **(A)** Full sample, **(B)** Team sport athletes, **(C)** Speed-strength athletes, **(D)** Precision-skill athletes. *Significant at.05 level; **at.01 level or lower; ***at.001 level or lower.

To examine whether time spent in the zone changed over time, data were binned into five bins. As illustrated in Figure 3A, there was a significant main effect of trial bin on proportion of time spent in the zone, *F*(4, 788) = 3.22, *p* = .012. *post-hoc* pairwise comparisons indicated that the proportion of trials in the zone was significantly greater in bin 3 compared to bin 1, *p* *=* .007, suggesting that athletes’ sustained attention gradually increased in the first half of the task. There was no main effect of trial bin on proportion of time spent in the zone for team sport athletes, *F*(4, 316) = 1.16, *p* = .328 (see Figure 3B), or speed-strength athletes, *F*(4, 132) = 1.45, *p* = .222 (see Figure 3C). However, there was a significant main effect of trial bin on proportion of time spent in the zone for precision-skill athletes, *F*(4, 332) = 3.54, *p* = .008. *post-hoc* pairwise comparisons indicated that the proportion of trials in the zone was significantly greater in bins 2 (*p* = .031) and 3 (*p* = .006) compared to bin 4 for precision-skill athletes (see Figure 3D).

## 4 Discussion

The overarching goal of the current study was to examine novel approaches to characterizing the sustained attention abilities of elite athletes. To do so, we first examined traditional indices of SART performance (i.e., speed, accuracy, variability) and their relation to sport type. Consistent with our hypothesis, team sport athletes demonstrated significantly greater accuracy on the SART compared to speed-strength and precision-skill athletes. However, inconsistent with our hypothesis, team sport athletes were not significantly faster or less variable than speed-strength or precision-skill athletes on the SART.

We then examined whether the VTC procedure could be applied to SART performance and, if so, whether it would enable a more nuanced examination of moment-to-moment fluctuations in sustained attention within individual athletes. Consistent with Esterman et al. (21) and other studies using the VTC procedure (28, 45, 46, 48, 49), our findings showed that participants were more variable, less accurate, and more error-prone when ‘out of the zone’ compared to when ‘in the zone’. Importantly, we provide preliminary evidence that this method can capture dynamic shifts in attention within individual athletes–an important consideration given that each sport and position impose distinct attentional demands (55, 56), and that each athlete inherently possesses unique attention abilities.

One potential mechanism underlying team sport athletes’ superior accuracy on the SART may include a heightened demand for inhibitory control. Specifically, playing team sports (e.g., rugby) depends on the use of enhanced inhibitory functions to filter out irrelevant stimuli (e.g., spectators yelling on the sideline) while simultaneously attending to relevant information, including sport-related stimuli (e.g., rugby ball) and each player's position on the field (57, 58). As such, the specific cognitive demands of team sports may train athletes to more effectively engage inhibitory control skills during task performance (18, 44, 59). Accordingly, accuracy, as opposed to mean RT or RT variability, may serve as a more sensitive indicator of SART performance for team sport athletes. In general, these findings highlight the importance of examining different behavioural indices when investigating cognitive functioning. This is particularly true for athlete populations, where the demands of their sport may foster specific cognitive skills (60) that are best captured by distinct behavioural indices.

Although RT variability has been increasingly recognized as a sensitive indicator of neuropsychological functioning (23), most IIV analyses have focused on between-group differences. Examining IIV at the group level may diminish its sensitivity by collapsing it into a single aggregate value, much like measures of central tendency, thereby obscuring moment-to-moment fluctuations that characterize an individual's sustained attention and cognitive functioning. To address this, we applied the VTC procedure to SART performance and demonstrated that attention fluctuates across multi-trial periods (i.e., epochs) of ‘in the zone’ and ‘out of the zone’ performance, consistent with patterns of sustained attention observed with the gradCPT (10, 21, 28, 45–49). However, in contrast to studies using the gradCPT, which indicate that the proportion of time ‘in the zone’ decreases over time, ‘in the zone’ trials were not always less prevalent in later trial bins in our study. When examining the proportion of trials ‘in the zone’ for the entire sample (Figure 3A), ‘in the zone’ performance increased from the beginning of the task (bin 1) to approximately the mid-point of the task (bin 3). The proportion of trials ‘in the zone’ then declined, but not to a significant degree. This may be because traditional continuous performance tasks, such as the SART, do not sufficiently tax participants’ ability to sustain attention over time (21). Specifically, the brief fixation periods between each trial may exogenously capture attention (61), thereby reducing reliance on the endogenous maintenance of attentional resources (21). As a result, participants may be less susceptible to shifting into ‘out of the zone’ states later in the task, despite the increasing demands on attentional resources that occurs over time. In addition, the SART is shorter in duration and presents stimuli at a slower rate (i.e., the SART includes 225 trials over approximately six minutes, whereas six minutes of the gradCPT constitutes 450 trials), thereby placing fewer demands on participants’ attentional resources near the end of the task.

Although the SART may not tax attentional resources to the same degree as the gradCPT, when examining the proportion of trials ‘in the zone’ by sport type (Figures 3B–D), distinct patterns of performance emerge. For team sport athletes, the proportion of trials ‘in the zone’ appears to be relatively stable throughout the task. Similarly, there was no significant difference in the proportion of trials ‘in the zone’ across trial bins for speed-strength athletes. However, the relatively small sample size in this group may have limited the statistical power required to detect such differences. Qualitatively, the pattern of results suggest speed-strength athletes may require more time on task before spending a greater proportion of time ‘in the zone’. In contrast, precision-skill athletes demonstrated a higher proportion of ‘in the zone’ trials in the first half of the task, which is consistent with prior studies using the gradCPT (28, 45–49) and suggests attentional resources may deplete at a faster rate in this group. Thus, although the SART may not tax attentional resources to the same extent as the gradCPT, differences in sustained attention over time remain evident when using analytic approaches, such as the VTC procedure, which more precisely capture fluctuations in attention over time. While the current findings are preliminary and further research is needed to clarify patterns of sustained attention across sport types and individual athletes, they provide early evidence that such patterns may reflect the unique attentional demands inherent to different sports. For instance, team sports (e.g., rugby) may require athletes to maintain attention and consistent athletic performance over extended periods of time, whereas precision-skill athletes (e.g., diving) may only need to engage focused attention for a brief, critical moment to succeed in their sport.

Superior athletic performance relies on efficient neural information processing (62). Long-term motor skill practice may induce neural plasticity across large-scale brain networks (63, 64), including the dorsal attention network (DAN), frontoparietal network (FPN), and default mode network (DMN), which are thought to play key roles in attentional control (59). The DAN, often referred to as the ‘task-positive’ network, is primarily engaged during goal-directed tasks that require attention and cognitive control (65, 66). In contrast, the DMN has predominantly been described as the ‘task-negative’ network, showing increased activity during periods of rest and task-unrelated thought (i.e., mind wandering) (67–69). Studies have shown that cardiovascular fitness has been linked to increased functional connectivity in the DMN (70). Likewise, the FPN supports attention, executive functioning, and motor control (59, 71–73), and evidence suggests athletes exhibit greater resting state connectivity between the FPN and distributed frontal regions (64). Researchers have hypothesized that negative associations between resting state regions may reflect the resolution of competing cognitive demands, and that anti-correlations between the DMN and task-positive networks are associated with improved cognitive performance (65, 74). Collectively, athletic participation engages multiple cognitive domains, including attention, which may in turn promote changes in brain structure, function, and connectivity (75, 76).

VTC analyses have been used to further elucidate the role of attention-relevant brain networks during sustained attention performance. According to the control model of sustained attention (77), brain regions associated with attentional control (FPN and DAN) and rest (DMN) should fluctuate, be inversely related, and accompany intraindividual variability in sustained attention performance. This model also posits that optimal performance is characterized by minimal communication between attentional control and DMN regions (19). Findings from studies employing the VTC procedure alongside functional neuroimaging techniques support this notion and indicate that greater DAN-DMN coupling is associated with more variable ‘out of the zone’ attentional states, suggesting that the dynamic push-pull relationship between these networks impacts sustained attention (21, 49). Moreover, participants exhibiting stronger DAN-DMN coupling across sustained attention task performance tend to show worse overall performance (10). Importantly, VTC studies have challenged traditional assumptions regarding the specific roles of the DAN and DMN during task performance. Specifically, ‘in the zone’ performance has been associated with greater DMN engagement, whereas ‘out of the zone’ states have been linked to increased activity within the DAN. This pattern suggests that ‘in the zone’ performance may reflect a more efficient and automatic mode of processing (i.e., akin to a flow state), while shifts into ‘out of the zone’ states may necessitate the recruitment of more reactive, task-positive control processes (21, 46). Together, these findings indicate that fluctuations between controlled (DAN) and rest/automatic (DMN) states correspond to variations in objective task performance (19), which are more precisely characterized using VTC analyses as opposed to traditional metrics (mean RT, accuracy, CoV) of sustained attention performance. Given that athletes have been shown to exhibit differences in resting state functional connectivity, it is plausible that similar differences in functional connectivity may also emerge during sustained attention tasks, where effective communication between DMN and task-positive/control regions is critical for optimal performance.

Findings regarding differences in cognition between athletes and non-athletes, as well as across levels of sport expertise, remain mixed. While some studies support the notion of superior cognitive abilities in athletes (3, 78–80), others have found no statistically significant differences between experienced and novice athletes (7, 18). Much of this work, however, has relied on between-group comparisons using single cognitive performance metrics, such as mean RT. In contrast, VTC analyses applied at the individual level may better capture the unique, moment-to-moment variability in cognitive performance.

VTC analyses may offer several advantages to studying the cognitive functioning of elite athletes. First, moment-to-moment RT variability has been linked to fluctuations in attentional stability, executive functioning, and arousal regulation (39). Performance variability has also been suggested to index the demands placed on top-down executive and attentional control (30), cognitive abilities that are critical for athletes who must maintain consistent performance under pressure (76). Research on the neural proficiency of athletes suggests athletic performance depends on athletes’ ability to flexibly shift between automated (i.e., ‘in the zone’) and reactive (i.e., engagement of DAN regions when ‘out of the zone’) attentional states in response to situational demands (81). Accordingly, for elite athletes who operate near the limits of perceptual and motor performance, intraindividual variability, rather than mean-based measures, may provide a more sensitive index of cognitive resilience, fatigue, or adaptability to changing demands. Second, expertise and long-term sport training are thought to engage and refine distributed neural networks underlying selective attention, sensorimotor integration, and cognitive flexibility (63, 64). These processes likely manifest not as static advantages but as dynamic modulations in performance stability over time. Capturing these fluctuations through VTC analyses may therefore illuminate how training history and task demands shape the temporal organization of cognition in elite athletes. Third, research involving elite athletes often faces constraints of small and heterogeneous samples, with participants differing in sport type, cognitive load, and training background. Under such conditions, within-person analytical approaches become particularly valuable. Intraindividual variance patterns in cognitive functioning may better allow researchers to disentangle idiosyncratic profiles of cognitive stability and adaptability, providing richer insights into how expertise is instantiated across individuals. Taken together, VTC analyses not only address methodological limitations of group-based designs but also align conceptually with the individualized nature of elite performance.

Several limitations and directions for future research should be considered. Sample sizes across sport types were not equal, and the relatively smaller sample of speed-strength athletes may have contributed to observed differences, or a lack thereof, on traditional performance metrics and in the proportion of time spent ‘in the zone’. Additionally, the present study proposes a novel framework for characterizing sustained attention in athletes, and future research should explore whether differences in functional connectivity among attention-relevant brain networks emerge when applying the VTC procedure to continuous performance tasks such as the SART. Future studies should also investigate whether moment-to-moment fluctuations in sustained attention performance correlate with sport-specific metrics, such as training history. Moreover, research should examine how variability in sustained attention changes over time and across stages of elite athletes’ cognitive development.

Taken together, the current study is the first to apply the VTC procedure to a traditional continuous performance task to examine intraindividual fluctuations in sustained attention among elite athletes. Given that continuous performance tasks, such as the SART, are commonly used in applied settings, demonstrating that VTC analyses can be successfully implemented within these paradigms may offer novel approaches for assessing cognitive performance in athletes. Elite athletes represent a specialized group in which specific sport characteristics (e.g., sport type, training history) place distinct demands on different dimensions of cognitive functioning. Accordingly, adopting within-person approaches to the study of athlete cognition is warranted and may advance the field toward more precise methods of assessment.

## Data Availability

The data supporting the findings of this study are not publicly available due to ethical and legal restrictions. The dataset contains sensitive information collected from athletes affiliated with a national sport organization, and its use is governed by institutional ethics approval and data-sharing agreements that strictly limit access to the approved investigators. These restrictions are in place to protect participant confidentiality, privacy, and organizational agreements, and therefore the data cannot be released upon request. Requests to access the datasets should be directed to Michelle Blumberg, mjblum@yorku.ca.
