# Supplementary material for: Sport-specific demands on sustained attention in elite athletes: a novel within-subjects approach for investigation
Source: Front Sports Act Living. 2026 Mar 13;8:1765258. doi: 10.3389/fspor.2026.1765258 (PMC13021763; doi:10.3389/fspor.2026.1765258)
Supplement: Supplementary file 1 [file Table1.docx]

**Supplemental Material**

**Table 1.** Predicting indices of sustained attention based on sport type with the precision-skill group coded as the reference group.

| Variable | *B* | *SE (B)* | *t* | *p* | *95% CI for B* |
| --- | --- | --- | --- | --- | --- |
| Model 1: Mean RT |  |  |  |  |  |
| Age | -0.10 | 1.59 | -0.06 | .949 | [-3.24, 3.04] |
| Sex: Female | 2.07 | 14.86 | 0.14 | .890 | [-27.25, 31.38] |
| Sport Type: Team | -4.13 | 15.87 | -0.26 | .795 | [-35.43, 27.17] |
| Sport Type: Speed-Strength | 1.50 | 20.74 | 0.07 | .943 | [-39.40, 42.39] |
| Model 2: d’ |  |  |  |  |  |
| **Age** | **0.09** | **0.02** | **4.83** | **<.001** | **[0.05, 0.13]** |
| Sex: Female | 0.08 | 0.17 | 0.47 | .637 | [-0.26, 0.42] |
| **Sport Type: Team** | **0.45** | **0.18** | **2.43** | **.016** | **[0.08, 0.81]** |
| Sport Type: Speed-Strength | -0.36 | 0.24 | -1.50 | .135 | [-0.84, 0.11] |
| Model 3: CoV |  |  |  |  |  |
| **Age** | **-0.01** | **0.003** | **-3.16** | **.002** | **[-0.02, -0.004]** |
| Sex: Female | -0.06 | 0.03 | -1.95 | .052 | [-0.11, 0.001] |
| Sport Type: Team | -0.01 | 0.04 | -0.23 | .820 | [-0.01, 0.05] |
| Sport Type: Speed-Strength | 0.06 | 0.04 | 1.60 | .112 | [-0.02, 0.14] |

Model 1: *F*(4, 193) = 0.02, *p* = .999, *R*^2^ = .001; Model 2: *F*(4, 193) = 7.78, *p* < .001, *R*^2^ = .14; Model 3: *F*(4, 193) = 4.16, *p* = .003, *R*^2^ = .08. RT = Reaction Time; CoV = Coefficient of Variation
